# Strategies in developing dimethyl sulfoxide (DMSO)-free cryopreservation protocols for biotherapeutics

**DOI:** 10.3389/fimmu.2022.1030965

**Published:** 2022-10-05

**Authors:** Marlene Davis Ekpo, George Frimpong Boafo, Jingxian Xie, Xiangjian Liu, Chuanpin Chen, Songwen Tan

**Affiliations:** Xiangya School of Pharmaceutical Sciences, Central South University, Changsha, China

**Keywords:** cryopreservation, cryoprotectants, biobanking, dimethyl sulfoxide, DMSO-free, biotherapeutics

## Introduction

Cryopreservation is a technology employed in long-term storage of biologics achieved by cooling to cryogenic temperatures (1, 2). This preservation technique has become increasingly relevant especially in the development and commercialization of cellular therapeutic products (3, 4). Conventional cryopreservation protocols involve the application of the permeating cryoprotectant; dimethyl sulfoxide (DMSO) due its ability to restrict ice nucleation and promote post-thaw viability (4). Although DMSO effectively preserves biologics, it can impair functional recovery (5–7) and induce a variety of mild to severe toxic effects in patients which must be avoided at all cost when administering immunotherapeutic products (8, 9).

The situation is considerably more critical during vitrification; a freezing method that has attracted heightened recognition as a faster and economic substitute to slow freezing as the unorganized liquid state of water is rapidly transformed to a glassy solid state without ice crystallization (10). In vitrification, high cooling rates and high concentrations (4–8 M) of cryoprotectants are usually required (11); enforcing on the exigency of using non-toxic cryoprotectants because increasing DMSO concentration is not advisable.

Efficacious cryopreservation and biobanking requires the development of safe and consistent storage protocols (12, 13). Favorably, such procedures should be devoid of xenogeneic or toxic components and to this effect, many scientists have put forward for the replacement of DMSO. Several groups have discovered/developed safer alternative cryoprotectants with a range of potential cryoprotection mechanisms like ice recrystallization inhibition, osmolality control, cell membrane stabilization and vitrification (14).

Numerous methods have since emerged to lessen the quantity of DMSO used (mostly by supplementation with other cryoprotectants) (15–20) or to completely eliminate DMSO application and these methods may also require special adjunct treatments, reagents or freezing protocols as would be discussed here in specifics. Attempts have also been made at preserving cellular products at non-freezing temperatures (21–25), advanceable by hypoxia and hypercapnia induced cytoprotection (26–28). Although highly beneficial to low-income countries where biobanking facilities are not obtainable, hypothermic storage is however limited to a few days thus fueling the need for safer freezing protocols.

But for the review by Weng et al. (29), studies on replacing DMSO are yet to be critically analyzed; a process that would track research accomplishments, expose novel supplementary techniques applied and encourage further research aimed at improving DMSO-free cryopreservation protocols for different biologicals. Through this article, we wish to draw the attention of researchers to possibility of a DMSO-free preservation era which is achievable in the nearest future. We provide prove of total exclusion of DMSO from cryopreservation solutions and summarize some of the supplementary techniques that have been applied to improve post-thaw viability and function. Our survey has also revealed the commercial availability of DMSO-free cryoprotectant solutions especially those used for cellular therapeutics, but there are limited studies to scrutinize or validate the potency of these products; which might be why most researchers are yet to largely patronizing products without DMSO as there is little to no evidence to back up these product claims. We therefore urge researchers to extend the application of these products to a wider range of biotherapeutics so as to speed up the availability of clinically approved products especially immunotherapeutics which is the answer to many complicated diseases like cancers.

## Challenges in cryopreservation with DMSO

Cryopreservation is an important determinant of the stability and activity of biopharmaceutical formulations. Several studies have proven the hypothesis that the application of DMSO can induce temperature-, time-, and concentration-dependent toxicities (7, 30). DMSO causes mitochondrial damage to astrocytes (31), and impacts negatively on cellular membrane/cytoskeleton structure and integrity by interacting with proteins and dehydrating lipids (32) as evident in the increased membrane permeability of erythrocytes (33) and altered chromatin conformation in fibroblasts (30). Also, the presence of DMSO in culture medium can induce unwanted stem cell differentiation (34).

Furthermore, repeated DMSO use even at sub-toxic levels can affect cellular epigenetic profile resulting in undesirable phenotypic disturbances (35). For instance, DMSO interferes with DNA methyltransferases and histone modification enzymes of human pluripotent stem cells causing epigenetic variations and reduction in their pluripotency (36, 37). Similarly, murine embryonic stem cells display disrupted mRNA expression levels of several markers following DMSO treatment (38).

Adverse reactions from cardiac, neurological, and gastrointestinal systems have been reported in patients receiving DMSO-containing cellular products (39, 40). These discoveries have led to the design of several washing procedures to ensure complete DMSO removal. However, the washing protocol usually involves agitation and osmotic/mechanical stresses which are to be avoided due to the fragile and sensitive nature of biologics post-thaw (40). The washing step can also be time consuming, expensive and resource wasting since a significant number of cells are loss in the process.

## Strategies in DMSO-free cryopreservation of biotherapeutics

Potent and safe alternative cryoprotectants to DMSO are highly desirable in order to meet the demands in the development and manufacturing of cellular and genetic therapies. In numerous instances, the observed cryoprotective effect is derived from a combination of two or more strategies as discussed below. These strategies and their outcome are also summarized in **Table 1**.

**Table 1:** Methods/Techniques used in DMSO-free cryopreservation of biotherapeutics.

Material	Cryoprotectant	Additional strategy	Outcome/Conclusion	Ref
Human umbilical cord matrix MSCs	1,2-propanediol and 1.0 M EG	Thawing *via* magnetic induction heating of magnetic extracellular Fe_3_O_4_ nanoparticles	Suppressed devitrification and recrystallization with improved cell survival	(41)
Mesenchymal stromal cells	100–300 mM sucrose	Addition of using 10% platelet lysate to expansion medium	Improved cryopreservation	(42)
Human dermal MSCs	Mannitol, lactose, sucrose, trehalose or raffinose	24-hour sugar pretreatment prior to cryopreservation	Cryopreserved MSCs has retained attachment, proliferation and multilineage differentiation	(43)
Human umbilical cord MSCs	Sucrose, trehalose and raffinose	Electroporation-assisted pre-freeze delivery of cryoprotectants	Improved cryopreservation of MSCs	(44)
HiPSCs	StemCell Keep™	Nano-warming	Improved cryopreservation of HiPSCs	(45)
HESCs	StemCell Keep™	N/A	Higher recovery rates and cell attachment	(46)
Human bone marrow-derived MSCs	Polyampholyte cryoprotectant	N/A	High viability and do not affect the biological properties of the cells even after 24 months of cryopreservation at 80°C.	(47)
HiPSCs	EG	-Dissociation of iPSCs with Accutase in the presence of a ROCK inhibitor -Programmed freezing	Up to 6-fold improvement in comparison to the standard freezing in clumps without ROCK inhibitor.	(48)
Wharton’ s Jelly Tissue	0.05 M glucose, 0.05 M sucrose, and 1.5 M EG in PBS	Programmed freezing	Higher post-thaw cell survivability	(49)
PDL cells and dental pulp tissues.	N/A	Programmed freezing using alternating magnetic field; “Cells Alive System”	Acceptable immediate autotransplantation results	(50)
Neural stem and progenitor cells	40% v/v EG and 0.6 M sucrose	N/A	Preserved expression of cell markers, proliferation and multipotent differentiation	(51)
HiPSCs	Sucrose, glycerol, isoleucine, and poloxamer 188	Controlled-rate freezing in a liquid nitrogen-based controlled-rate freezer	Improved cryopreservation of hiPSCs	(52)
Erythrocytes	0.1 wt% PVA	N/A	Significantly high post-thaw cell recovery	(53)
Mesenchymal Stromal Cells	Osmolyte-based freezing solutions	N/A	Comparable post-thaw recovery and improved post-thaw attachment	(54, 55)
MSCs	Amphiphilic Block Copolymer	N/A	Excellent MSC proliferation and multilineage differentiation properties	(56)
Human bone marrow-derived MSCs	2 M 1,2-EG, 2 M 1,2- propyl alcohol, and 0.5 M trehalose	Nano-warming with synthetic Pluronic F127-liquid metal nanoparticles (PLM NPs)	Threefold increase in viability, and maintained attachment, proliferation, surface marker expression, and multilineage differentiation	(57)
Human MSC monolayers	6.5 M EG, 0.5 M sucrose, and 10% w/w COOH-PLL	Slow vitrification at rates of 4.9 and 10.8°C/min	Significantly improved viability with less apoptosis	(58)
Natural killer cells	Poly-L-lysine, Ectoine, dextran and sucrose	N/A	Maintained cells viability, morphology and cytotoxic activity following long-term cryopreservation up to 2 months.	(59)
Erythrocytes	Biomimetic Block Copolymer Worms with PVA	N/A	Improved cell recovery with no evidence of hemagglutination or abnormal cell morphologies.	(60)
Human ADSCs	1.0 M Trehalose and 20% glycerol	N/A	High preservation efficiency with acceptable outcomes	(12)
Human ADSCs	Trehalose	Nanoparticle-mediated intracellular delivery of trehalose	Eliminates multistep washing of the cryopreserved cells to remove toxic/penetrating cryoprotectants	(61)
HiPSCs	Trehalose-based cryosolutions containing EG or glycerol	N/A	High cell viability and high stability with retained their morphology, self-renewal, pluripotency and differentiation.	(36)
HSCs	HP01 (Macopharma)	N/A	Conserved full short- and long-term post-thaw cellular activity	(62)
HiPSCs	Sucrose, glycerol, L-isoleucine, poloxamer 188 (P188)	N/A	Highly viable and functional HiPSCs	(5, 63)
HSCs	Antifreeze protein mimetics produced by X-Therma Inc. (Richmond, CA), XT-Thrive A™ and XT-Thrive B™	N/A	X-Therma formulations may offer clinically safer alternative to DMSO-based solutions.	(64)
HSCs, T-cells, CD34^+^ cells	Pentaisomaltose™, CryoScarless™, CryoNovo P24™, CryoProtectPureSTEM™	N/A	Comparable results to those cryopreserved with DMSO	(9, 65–67)

N/A, Not Applicable; HiPSCs, Human induced pluripotent stem cells; Human embryonic stem cells, hESCs; EG, Ethylene glycol; DMSO, Dimethyl sulfoxide; PDL, Periodontal ligament; PVA, Polyvinyl alcohol; COOH-PLL, carboxylated poly-L-lysine; HSCs, Hematopoietic stem cells; MSCs, Mesenchymal stem cells; ADSCs, Adipose-derived stem cells.

### Alternative cryoprotectants to DMSO

Replacing DMSO with other cryoprotectants is the typical approach in eradicating the use of DMSO in cryopreservation. Kuleshova et al. vitrified neural stem cells using a combination of ethylene glycol (EG) and sucrose. Post-storage evaluations revealed no substantial differences between fresh and vitrified cells in cell markers expression, proliferation or multipotent differentiation (51).

Osmolyte-based freezing solutions containing varying blends of sucrose, glycerol, creatine, isoleucine and mannitol have supported the recovery and survival of mesenchymal stromal cells when compared to conventional preservation with DMSO. These solutions conferred cryoprotection, retained cell differentiation capacity and modulated the cytosine-phosphate-guanine epigenome (54). StemCell Keep™ has been proven effective for the cryopreservation of human induced pluripotent stem cells (hiPSCs) (68), human embryonic stem cells (hESCs) (46) and mesenchymal stem cells (MSCs) (47). Further investigation on the mechanism of cryoprotection showed that the polyampholyte is adsorbed on to the cell membrane suggesting that it can confer protection on cell surface and eliminate the use of proteins and DMSO (47). Other polyampholytes have also shown great potential as DMSO substitutes in storage of murine L929 cells and rat MSCs (69), natural killer cells (59) and other mammalian cell types (70).

A cocktail of non-toxic and Food and Drug Administration (US-FDA)-approved infusible substances including sucrose, glycerol, isoleucine, human serum albumin, and poloxamer 188, have been applied for preservation of hiPSCs (52). In a recent study by Park et al., a block copolymer; PEG−PA (5000−500) has been presented as an excellent cryoprotectant where the recovered stem cells exhibited acceptable survival, proliferation and multilineage differentiation post-thaw (56).

These studies and more presented in **Table 1** portray the synergistic activity of several biocompatible cryoprotectants and suggests that this approach may be an innovative paradigm for safe cryopreservation. Presently, commercially manufactured DMSO-free cryosolutions like Pentaisomaltose™, CryoScarless™, CryoNovo P24™, StemCell Keep™, CryoSOfree™ and XT-Thrive™ are available but their quality and capacity to protect a wide range of therapeutics is under-investigated. Further research would involve testing the compatibility of these cryoprotectants with other biotherapeutics. In addition to substituting cryoprotectants, supporting techniques used to improve on solvent-free cryopreservation are discussed below.

### Pre-cryopreservation treatment

The pretreatment of biotherapeutics with cryoprotective and stabilizing agents prior to cryopreservation is largely becoming a viable approach to ensuring safe storage. Sugar pretreatment, supplementation of expansion medium with 10% platelet lysate and slow freezing is reportedly an effective protocol in DMSO-less cryopreservation of adipose-derived stromal cells (42). Similarly, sugar pretreatment increased survival, metabolic activity, attachment, proliferation and multilineage differentiation after recultivation of dermal MSCs (43). Improved results are also obtained when the sugars are positioned intracellularly as performed by Mutsenko and coworkers who explored electroporation-aided delivery of cryoprotective sugars in human umbilical cord MSCs (44). Also, pre-incubation of MSCs with osmolyte-based freezing solutions could foster effective cryopreservation (54). These results corroborate the potential advantages of pre-cryopreservation treatment(s).

### Programmed freezing methods

Programmed freezing offers improved control over ice nucleation parameters. A technique involving a magnetic field driven freezer termed “Cells Alive System” has been used to prevent formation of intracellular ice for up to three months. The magnetic field vibration function prohibits water molecules from creating clusters during freezing. Although the optimal conditions needed for survival and viability of isolated human periodontal ligament cells (PDL), pulp tissue and tooth using CAS freezers were determined previously (71) (72) with DMSO, the technique proved equally effective without DMSO, promoting greater survival rates over that obtained with conventional freezers (50). Programmed freezing has also been used for cryopreservation of human Wharton’s Jelly Tissue, showing higher post-thaw cell survivability when used in conjunction with a freezing solution consisting of 0.05 M glucose, 0.05 M sucrose and 1.5 M EG in PBS (49).

Matsumura et al. reports a simple, novel slow vitrification method at 4.9 and 10.8°C/min for the cryopreservation of MSC monolayers using a polyampholyte based vitrification solution. Thermal analysis confirmed stable vitrification and post-thaw assessment revealed significantly improved viability and retained differentiation capacity (58).

### Thawing protocol

Due to the low thermal conductivity of biological samples, the conventional approach of rewarming large-volume cryopreserved samples in a water bath heated at 37°C is associated with non-uniform distribution of temperature, which can induce thermal stress (41). Preferably, a high heating rate is desirable during thawing, because devitrification and recrystallization may occur if the temperature cannot be elevated rapidly above the sample’s melting point (73). Therefore, both the heating rate and uniformity of heating during rewarming are important to cryopreservation especially vitrification. Nevertheless, attaining the optimal rewarming rate remains a major factor complicating effective vitrification.

Wang et al. proposes magnetic induction heating (MIH) of extracellular Fe_3_O_4_ magnetic nanoparticles also called nano-warming technology as a method to amplify rewarming. This technique was successfully applied to rewarming vitrified MSCs where the sample was thawed by plunging the straw into a 0.2 M trehalose supplemented culture medium heated to 37°C. Then, the system was subjected to MIH under alternating magnetic field at a medium frequency for a duration of 10 s. Results obtained reveals the prospective benefits this technique holds as it significantly hindered ice recrystallization/devitrification during rewarming and improved cell viability (41). More recently, Ito et al. also employed the nano-warming technology for thawing of hiPSC using StemCell Keep as cryoprotectant. Similarly, nano-warming showed more uniform and rapid rewarming of vitrified samples, prevented devitrification/recrystallization and improved viability (45).

Another nanotechnology assisted thawing approach involves the utilization of soft liquid metal nanoparticles possessing reproducible photothermal stability, high photothermal conversion efficiency, low cytotoxicity and the ability to suppress ice formation. This technique promotes less ice nucleation during freezing and ultrarapid rewarming while thawing. Human bone marrow stromal cells have been successfully rewarmed with this technique (57).

These studies reveal that the advantages of nanotechnology can be capitalized on to promote safe rewarming post-cryopreservation after verifying the biocompatibility of the nanoparticles.

## Conclusion

A critical step prior to the clinical application of biotherapeutics is the optimization of cryopreservation protocols that minimize post-thaw alterations in the stability and potency of preserved materials. Cryosolutions containing 10% of DMSO is a widely used cryopreservative but there is an increasing amount of evidence showing inconsistent results on its impact on post-cryopreservation performance of biologicals. This drawback forms the basis for the development of safer preservation protocols. DMSO-free strategies have the potential to alleviate the aforementioned obstacles as demonstrated by studies discussed in this article. The application of other cryoprotectants, combined with other techniques like programmed freezing, pretreatments and modified thawing protocols have shown good prospects.

In conclusion, the development of effective DMSO-free cryopreservation techniques that will provide high post-thaw viability and preserve original morphology and functioning remains key because this is essential to hastening the industrialization and clinical application of biotherapeutics. We are of the opinion that more research efforts should be put into the development and performance validation of trademarked DMSO-free products. In cases where DMSO elimination is unavoidable, only confirmed safe concentrations should be applied preferably in combination with non-toxic cryoprotectants and other potent strategies.

## Author contributions

Conceptualization: ME, CC, ST. Writing—original draft preparation: ME, JX, XL, GB. Writing—review and editing: ME, XL, GB, CC and ST. Supervision and approval: ST. All authors contributed to the article and approved the submitted version.

## Conflict of interest

The authors declare that the research was conducted in the absence of any commercial or financial relationships that could be construed as a potential conflict of interest.

## Publisher’s note

All claims expressed in this article are solely those of the authors and do not necessarily represent those of their affiliated organizations, or those of the publisher, the editors and the reviewers. Any product that may be evaluated in this article, or claim that may be made by its manufacturer, is not guaranteed or endorsed by the publisher.
